# Artificial Fecundation, &c.

**Published:** 1889-06

**Authors:** 

**Affiliations:** Paris, France


### ARTIFICIAL FECUNDATION AND POSITION DURING COITION.

BY DR. LUTEAUD, PARIS, FRANCE.

Gentlemen :—Artificial fecundation had been tried on the inferior animals during the century before this, but Swammerdam and Roesel did not have any success with it, and it was the naturalist Spallanzani, in 1767, who made the first successful experiments in dogs. The prejudices of that time prevented him from trying it on man, although he several times expressed his belief that it was possible to produce fecundation in human beings as well as in animals. The first success in man we know of was made by Hunter, in 1799. The famous surgeon gave a consultation to a man who was afflicted with hypospadias, and wished to become a father. He was advised to inject the sperm, which he could only deposit in the vagina, and the injection was followed by a complete success. Girault gave the first authentic cases of artificial fecundation; which he obtained by injecting the fluid into the uterus, using a urethral sound and blowing the sperm through it. In 1869, he published twelve observations, the first of which dated from 1838; twenty-seven injections were made in all and only two cases failed, while none of them had any complications. Marion Sims, in 1866, related a case of success, and the same year Gigon published a thesis on the subject with several observations. In 1871, Courty gave a short account of the operation, and Prof. Pajot now recommends it. He thinks it is advisable, *after all other means have failed*, and he proposes for the operation a new instrument, which he calls a "*fecundateur*," (which consists of a gutta percha sound terminated by a little sack or ampulla). Pajot advises artificial fecundation not only in the case of malformation in man, and in uterine deviation in woman, but also in sterile unions, where the spermatozoids are not found imperfect or wanting. Leblond, in his treatise on gynecological operations, gives this operation; so that it may be said, in fact, that all the classical authors admit its performance. Dr. Luteaud believes, with Pajot, that it is only to be tried after all other methods have failed. This is a fundamental point, but it is to be asked, all the same, if it might not be advised before performing amputation of the os, which, if it does not constitute a real danger to life, still is a traumatism that has certain dangers. This question may be left to a certain extent to the person most interested. There are women who prefer an operation to simple injection into the uterus, owing to the shock given to their modesty, which is perfectly legitimate, but these remarks do not apply to those cases where the operation of amputation is advised for other reasons than simple sterility.

It is understood that artificial fecundation is not to be attempted *after the meno-pause*, and it is also contra-indicated in cases of malformation of the pelvic or genital organs in woman, and again in atrophy of the uterus. The existence of chronic pelvi-peritonitis gives but little chance of success, and again metritis and endometritis, with all the inflammatory uterine troubles, will prevent conception, unless they are treated and cured first of all. The operator must be certain that the fecundating fluid contains spermatozöids, for while we know but little of the pathology of these corpuscles we do know that sperm without them is useless; the microscope must then be used first. Then there are several *moral* considerations for the physicians to consider. If there is a tubercular, cancerous, or even scrofulous diathesis present in one or both of the parents, ought he to advise progeny? No! Nature herself often refuses in such cases, as there may well be imperfect beings created. A first condition of success is that the menstrual molimen exists, and as said before, that the spermatozoids are present, while no malformation opposes conception and delivery. Modern physiological researches tend to prove that fecundation in women takes place before the menses rather than after the period, so that it is advisable to attempt artificial fecundation during the three or four days before the menses are expected; then, if there is a failure it can be tried again during the week after the period.

As to the method of operation: A large number of plans have been proposed, but only these will be mentioned which are compatible with the dignity of a physician and the modesty of woman. Marion Sims's operation is well known in America. It consists of an injection with a glass syringe with a rounded bulb on the end, and he insisted that only one drop be injected, as he feared that the uterus would contract and expel any larger quantity, but it has since been found that the uterus will retain two or three drops. Courty's method is something the same, except that he collected the fluid by advising the use of a condom cover to retain it. Professor Pajot's method consists in a syringe arranged to collect the sperm by aspiration and injecting it into the os some two or three centimetres. He first used the touch to find the os, but now operates *de visu*. Four or five attempts are as many as it is worth while to try, these to be done at the menstrual periods as mentioned. Any physician who is accustomed to passing a sound into the uterus, can do it. The most important point is in regard to the cervical canal. The operator must assure himself that it is permeable and also see what its direction is, so that at the last moment he will not have to lose time. This information should be secured some time ahead, and it is also well, beforehand, to institute a treatment of the vaginal mucous membrane. It is now well known that the vaginal secretions are often acid and that this condition is almost surely destructive to the spermatozoids. So that a treatment by alkaline injections should be commenced a week or more beforehand. M. Luteaud recommends a solution of nitrate of potash ($\frac{1}{1000}$) morning and evening injections. The husband must be advised to abstain for a certain time, so that the quantity of the secretion will be secured. A basin is prepared with hot water (40°, centigrade) and a Ferguson speculum with a special form of intra-uterine sound is placed in it. Then the moment the coition is over, the operator is introduced, when by *Luteaud's* method, he simply passes the speculum, which collects the liquid by pushing it towards the os uteri. The special sound is then introduced with a rubber bulb on it, a few drops of the liquid is aspirated, and at once injected into the uterus. A small vaginal tampon of borated cotton is passed, to be left against the os for a few hours, and the patient is advised to remain in bed during that time.

M. Luteaud gives his statistics. He practiced artificial fecundation in twenty-six cases, in women from twenty-seven to thirty-nine years of age. He only claims relatively little from it, for he had only two cases of entire success; besides one of hypospadias, and two other cases of incomplete success, where there had been conception produced, but abortion came on within three months. At the same time he feels that it is an operation that should be advised in the cases mentioned.

*Position during Coition.*—Dr. Luteaud concludes his lectures by some practical considerations on coition. which, as he says, is an impetuous act accomplished under the influence of an instinctive impulsion, without reflection, and in certain cases a physician should study the conditions under which it takes place so that he can give the interested parties good scientific advice that may allow them to succeed in the reproduction of the species. This result can sometimes be attained by a careful study, based on physiology and on the situation of the organs in the female, which are often so modified, that it is difficult if not impossible for the sperm to penetrate the cervical canal. The object of coition, practiced for fecundating purposes, is to introduce sperm into the uterus, or at least, to *project* it against the os uteri; and a doctor, consulted by a sterile woman, should inquire if coition has been so practiced that that object could be obtained. He should also examine if the male organ is properly formed, if the os uteri is in the axis of the vagina, and if there are no false vaginal passages. Finally, he should see if there are not certain positions or attitudes that he could advise in the case given, that would produce the result desired, that is, to place the organs under the most favorable conditions for fecundation. Certain defective male organs are more frequent than we think (epispadias and hypospedias), and the physician should always examine the husband. Pajot has often insisted upon the *false routes* that may be created in the vagina, owing to deviations of the uterus, or to a conical disposition of the os, and, above all, to a hypertrophy with elongation of the uterus, which allows the male organ to penetrate into one of the vaginal *cal-de-sacs*. The sperm is then destroyed by the acidity of the secretion, and conception is prevented. This goes on, for coition being usually performed in a uniform manner, the false route becomes a permanent one, and becomes a cause of infecundity that only increases with time. Uterine deviations are the principal cause that most often place the mouth of the organ out of the axis of the vagina. These are so well known that it is well to inquire if it is not possible to remedy these defective conditions by advising *certain positions during coition*, which may bring the glans penis in more direct contact with the os uteri.

In the human species sexual intercourse takes place, as a rule, in *dorsal decubitus*. The vertical transformation of man, which has taken place so gradually, did not, however, allow any extra accommodation to the uterus for the present upright position of woman, and the organ keeps the identical situation that it has in monkeys and other quadrupeds. It is normally in anteversion, and forms an angle with the vagina, which makes a most unfavorable condition for fecundation, according to the method of coition as practiced by our race. It results from this, that the position *more canum* is the most favorable one for conception. This is confirmed by clinical experience and by a mechanical study of the organs. One has only to place a patient in the genu-pectoral position and apply the Sims' speculum to see how easily the os is reached in such a position. Not only is it more accessible, but it also changes the situation so that it, for the moment, at least, cures the retroflexion and the vaginal false passages that may have existed.

It is, therefore, well to recommend the position *more animalium*, above all in cases of retroflexion. Hegar also advised the husband to raise up slowly the abdominal mass, and let it go suddenly at the end. No matter how repugnant to the feelings of a woman such advice may be, it is certain that such is the desire of sterile women to have children that they will gladly submit to the modification of position; few but those who have had experience of it can understand the intense desire they have for maternity. M. Luteaud states that the female seated upon the male during coition brings the organ also into the same favorable position, and has the advantage of allowing the position to be kept for a time *post-coitum*, which is favorable for conception. Dr. N. Gueneau de Mussy advised the husband to practice certain manipulations to facilitate the entrance of the sperm into the canal of the uterus.

Sed aud illicitum mihi visum est, si post diversatentamina dicitius uxor infeconda manserit, ipsum maritum digitum post coitum in vaginam immitere, et ita receptum semen uteri ostio admovere. Et cum ostiolo uteri hœret, ut in pervium canalem, spermatozoidum motibus faventibus, prodeat sperare non absurdum.

These latin directions may be useful in certain cases, but they would first require all husbands to practice the touch and be able to recognize the os uteri. Prof. Pajot advises who have anteversion to *retain their urine* for some time before coitus, and when there is a retroflexion he brings on constipation, to fill the lower intestine and throw the uterine mouth forwards. In lateral versions the position on the side can be recommended.

In regard to the most favorable time for conception, M. Luteud advises four days before and one week after the menstrual period; but he says that it cannot be contested but that it takes place at all times of the month. He also is inclined to believe that frigidity or a passive action in women is an obstacle to fecundation, and that the orgasm in them is useful for conception, but not actually necessary, for it cannot be denied that the conception takes place without it. But both complete erection and spasm opens the cervical canal and leads to the desired object better that passive action on the part of women. —*Times and Register.*

